# Determination and Assessment of the Toxic Heavy Metal Elements Abstracted from the Traditional Plant Cosmetics and Medical Remedies: Case Study of Libya

**DOI:** 10.3390/ijerph16111957

**Published:** 2019-06-02

**Authors:** Aiman M. Bobaker, Intisar Alakili, Sukiman B. Sarmani, Nadhir Al-Ansari, Zaher Mundher Yaseen

**Affiliations:** 1 Chemistry Department, Faculty of Science, University of Benghazi, Benghazi 16063, Libya; aiman.bobaker@uob.edu.ly (A.M.B.); intisar.alakeili@uob.edu.ly (I.A.); 2 School of Chemical Sciences and Food Technology, Faculty of Science and Technology, Universiti Kebangsaan Malaysia, Bandar Baru Bangi 43600, Malaysia; sukiman.sarmani@gmail.com; 3 Civil, Environmental and Natural Resources Engineering, Lulea University of Technology, 97187 Lulea, Sweden; nadhir.alansari@ltu.se; 4 Sustainable Developments in Civil Engineering Research Group, Faculty of Civil Engineering, Ton Duc Thang University, Ho Chi Minh City, Vietnam

**Keywords:** traditional plant cosmetics, toxic heavy metals, henna, walnut tree bark, ICP-MS, Libya

## Abstract

Henna and walnut tree bark are widely used by Libyan women as cosmetics. They may contain lead (Pb), cadmium (Cd) and arsenic (As), which, in turn, pose a high risk to their health. This study aims to determine the levels of Pb, Cd and As in henna and walnut tree bark products sold in Libyan markets. The products were analyzed for their Pb, Cd and As content by using inductively coupled plasma mass spectrometry (ICP-MS) after a microwave acid digestion. The results showed a significant difference between the henna and walnut tree bark samples in terms of their heavy metals content (*p* < 0.05). The highest heavy metal concentrations were observed in the walnut tree bark samples whereas the lowest was observed in the henna samples. In addition, 60% of the henna and 90% of the walnut tree bark samples contained Pb levels and approximately 80% of the henna and 90% the walnut tree bark samples contained Cd levels, which are much higher than the tolerance limit. However, As concentrations in all the samples were lower. The results indicated that such cosmetics expose consumers to high levels of Pb and Cd and hence, to potential health risks. Thus, studying the sources and effects of heavy metals in such cosmetics is strongly recommended.

## 1. Introduction

Henna and walnut tree bark, locally known as souak, are traditional plant cosmetic products that have been widely used in North Africa, the Middle East, and Western Asia countries for medical and cosmetic purposes [1,2,3,4,5]. As these traditional plant cosmetics are not permanent, painless, and cheap, and carry no risk of HIV or hepatitis infections, they are extremely popular among women as cosmetics [6,7,8,9].

Henna is sourced from the finely pulverized dried leaves of *Lawsonia inermis* L. plant; this tree is grown in the tropical and subtropical regions of North Africa, India, Sri Lanka, and the Middle East [10,11,12,13]. As per ethnobotanical and historical information, henna is among the first plants to be used for cosmetic purposes [2,3,4,14,15]. Henna contains an active dye (red-orange pigment), lawsone (2-hydroxy-1,4-naphthoquinone), which can be used to color hair and skin [1,6,16,17,18,19,20,21]. Traditionally, it is used as a medicine to remedy several ailments [22,23,24,25,26,27,28]. Henna has several biological activities [12,29,30,31,32,33,34].

When used for cosmetic purposes, henna is often mixed with water or oil to make a paste, which can be applied to the skin or hair. Upon the application of the henna paste on the skin, its dye (lawsone) will be adsorbed to the outermost skin layer, creating a red-orange stain [35]. Marketed henna is often mixed with various herbs and different chemical additives (which contains high levels of trace elements) to make it permanent or stronger [1,36,37,38,39,40]. Natural henna is generally safe and well-tolerated in humans, but henna with additives may have side effects or even life-threatening effects [37,41,42].

Henna is extremely popular in Libya as it is part of the culture and traditions. Various henna products with various colors are available in the Libyan market. Some of them are locally produced, while some are imported from Sudan, Yemen, Tunisia, India, and Pakistan. Unfortunately, over the last 10 years, henna has become a life-threatening material for Libyan women, especially in the eastern region of Libya where more than 550 victims of henna poisoning have been reported, of which 57 died as per the Libyan Ministry of health. Till today, the scientific information about the real reasons that led to this wide scale poisoning and death cases remain unknown. Despite the seriousness of the problem, published studies on henna in Libya are rare [43].

Walnut tree bark, locally known as souak, is another traditional plant cosmetic material widely used to clean and whiten teeth, which, after use, leaves an orange-brown tint on gums and lips. It is obtained as a natural chewing stick from the bark of a walnut tree (*Juglan regia* L.) [8,9]. Additionally, it is used as a medicine, improves dental hygiene [44,45,46], and widely applied in pharmaceutical industries [5,47,48,49,50,51,52]. Walnut tree bark is not locally produced in Libya, but imported from other countries like Algeria, Morocco, Tunisia, Sudan, and India. It is available in herbalist shops as a solid tough and fibrous material in thin pieces or shreds of varying lengths. Walnut tree bark has a feeble order and somewhat acrid bitter taste. Currently, it is extensively used by Libyan women as toothbrush and dye for aesthetics. They detach a piece of walnut tree bark and rinse it with water, then, rub it to their teeth and gums for a few minutes before rinsing their mouth with warm water several times. The process can be repeated more than twice a week. Moreover, walnut tree bark is among the popular materials added for intensifying the color of henna.

Heavy metals toxicity to human is well documented [53]. Lead (Pb), cadmium (Cd) and arsenic (As), which are extremely toxic to humans, accumulate in internal organs over time [54,55,56,57]. The human skin has been reported in a study to absorb soluble Pb as evidenced by its increased concentration in sweat, blood, and urine within 6 h of its application on the skin [58]. Cd is a human carcinogen as per the World Health Organization [59]. Arsenic, on the other hand, has a high affinity for keratin and as such, can be easily found in the hair and nails. Acute overexposure to arsenic results in skin eruptions, nails striation, and alopecia [60].

The maximum amounts of metals present in cosmetic ingredients provided by Cosmetica Italia are 20, 5 and 5 for Pb, Cd and As, respectively. Dermal absorption of As is 1.9%. The extent of percutaneous absorption of As was found to be 6.4% for trace doses and 2.0% for high doses in monkeys, and 1.9% when applied in trace doses onto human skin samples. Dermal absorption of Cd is 0.8%; about 0.3–0.8% of Cd is absorbed through the skin. Dermal absorption of Pb is 0.3%. The percutaneous absorption of lead acetate following the use of hair coloring preparations was almost nil, ranging from 0 to 0.3% of the dose applied onto the skin. Moderate absorption was detectable in the case of the damaged epidermis [61]. In a study conducted by Ibrahim and others in 2016, they investigated heavy metal content in henna and reported that the mean concentration of these metals was higher than the permissible levels [62].

Unfortunately, international standards for impurities in cosmetics are currently unavailable. However, the limits provided by the United States Food and Drug Administration (US FDA) for Pb and As contents of henna (as a color additive for cosmetic products) were 20 and 3 μg·g^−1^, respectively, while the Canadian regularity limits for certain metals in cosmetics were 10 μg·g^−1^ for Pb; 3 μg·g^−1^ for As, and Cd. Other legislation, such as the Danish Statutory Order on Cosmetics number 489, has banned the marketing of all cosmetic products containing Pb, Cd, and, As.

Many traditional cosmetic and medical plants play an important role in the general state of health of a population [63,64,65,66,67,68] and can pose health risks due to the presence of heavy metals [1,69,70,71]. Therefore, henna and walnut tree bark may be considered as a potential source of heavy metals poisoning as their intensive use may increase the body intake of heavy metals through percutaneous absorption.

Given the increasing concern about the use of henna in the Libyan society and the absence of any legal control of heavy metals in traditional plant cosmetics sold in the Libyan markets, we aim to investigate whether traditional plant cosmetics sold in Libya are possible sources of toxic heavy metals. Our study will contribute to the existing body of knowledge in this area. Therefore, the aims of this study are as follows: To estimate Pb, Cd and As contents in henna and walnut tree bark traditional plant cosmetics sold in Libyan markets, and to compare the obtained results with internal standards for heavy metals in cosmetic products.

The main enthusiasm for the current research is to have an informative vision of the heavy metal concentrations (i.e., Pb, Cd, and As) in the henna and walnut tree bark in Libya. Indeed, the accurate determination of heavy metals in cosmetics is important as they have a narrow range of safety between adequate amounts and excessive consumption. Among the various methods proposed for heavy metals analysis in cosmetic products, inductively coupled plasma mass spectrometry (ICP-MS) is the most frequently used due to its relative simplicity, low sample volume requirements, and low detection limits [72,73]. A method using microwave acid digestion, followed by ICP-MS determination for the analysis of heavy metal content in the henna and walnut tree bark samples was used for this study.

## 2. Material and Methods

### 2.1. Chemical and Reagent

All chemicals and reagents used were of analytical-reagent grade with no further purification. Nitric acid (HNO_3_) 69% (w/v) and hydrogen peroxide (H_2_O_2_) 30% (w/v) purchased from Merck (Darmstadt, Germany) were used for sample digestion. Ultrapure water (18.2 MΩ.cm quality, model: LPTA/PB/7/1-Maxima Ultrapure Water, ELGA company, Milan, Italy) was used for solution preparation and dilution. The heavy metal standard solutions used for the calibration were prepared by the step dilution of certified stock solutions of 1000 mg·L^−1^ of each heavy metal (Pb, Cd and As) purchased from Merck (Darmstadt, Germany). For the accuracy tests, a certified reference material (CRM) bush branches and leaves (NCS DC 73,348 CRM) from the China National Analysis Center for Iron and Steel (Beijing, China) were used.

To avoid sample contamination, all glassware, plastic ware, and digestion vessels were washed with Extran^®^ detergent, soaked in 10% (v/v) HNO_3_ for a minimum of 24 h, washed several times with tap water, rinsed thoroughly with ultrapure water, dried and stored in closed polypropylene containers until use.

### 2.2. Sample Collection

Convenience sampling technique was implemented in 2018 to collect only henna and walnut tree bark (souak) products that are widely used by Libyan women as traditional plant cosmetics. Samples were collected from well-known local herbalist shops who were conveniently available to participate in this study. These herbalist shops sell traditional plant cosmetic products imported from different countries and those manufactured locally. The sample size ensured that a maximum number of different brands of henna and walnut tree bark products sold were obtained. Notably, all the walnut tree bark products are sold as pieces of plant bark in open polyethylene containers without information labels. Meanwhile, all the henna products are sold without information about their chemical contents.

A total of 15 different brands of powdered henna and 10 unpacked walnut tree bark (souak) samples were purchased from local herbalist shops in Benghazi City, Libya. From each brand of henna and walnut tree bark type, five samples were collected, i.e., 75 and 50 different samples of henna and walnut tree bark (souak) were collected, respectively. About 15 g of each henna brand were separated by quartering sampling method (CAC, 2004) and collected separately in polyethylene ziplock bag previously washed with 10% (v/v) nitric acid (HNO_3_) and ultrapure water and dried at room temperature before the sample collection. The brand names of the powdered henna were blinded, labeled and given the codes H1–H15. A 200 g of each unpacked walnut tree bark samples of each brand were dried, ground and mixed together. Likewise, 15 g of powdered walnut tree bark samples were separated by quartering sampling method (CAC, 2004) and collected in a pre-cleaned polyethylene ziplock bag, labeled and given the codes S1–S10. The collected samples were kept at room temperature under non-humid conditions until analysis. Analyses were completed within 1 month after collection.

### 2.3. Samples Pre-Treatment

Prior to analysis, the henna samples were dried at 105 °C in an air ventilated oven for 24 h. The walnut tree bark samples were cut into small pieces, dried at 50 °C in the oven for 48 h, grounded into a fine powder using a mixer grinder, and then sieved through a 250 μm mesh. The obtained powdered walnut tree bark samples were again oven-dried for 24 h at 105 °C. Both dried henna and walnut tree bark samples were allowed to cool over silica gel and stored separately at room temperature in tightly closed Zip lock polyethylene bags until analysis.

### 2.4. Sample Digestion

Approximately 0.2 g of each dried henna or walnut tree bark sample was accurately weighed in a dry and clean digestion vessel. Then, 10 mL of HNO_3_/H_2_O_2_ mixture (v/v, 3:1) and 4 mL of ultrapure water were added to the sample [74]. The digestion process was performed at room temperature for about 15 min to ensure the completion of the initial reaction. Later, the digestion vessel was moved to a laboratory microwave digestion system for the microwave-assisted digestion, as shown in Table 1. After digestion, the vessel was cooled to room temperature before being opened and sonicated for 30 min at room temperature to eliminate nitrous oxide vapor. The inner side of the vessel lid was rinsed with ultrapure water before transferring the digested solution quantitatively into a volumetric flask using 1% HNO_3._ The final volume of the solution was made up to 100 mL using ultrapure water before filtering the samples through a 0.45 μm Millipore membrane syringe filter. The samples were later stored at room temperature in polyethylene bottles and analyzed for heavy metals contents by ICP-MS within a week of storage. The measurements were performed in triplicate, and the mean value was calculated on a dry weight basis (µg·g^−1^, dry weight). The blank solutions for the heavy metals determined by ICP-MS were prepared by following all the analytical steps of the proposed method in the absence of any sample or standard. A developed Perkin Elmer method was used for the calculation of the limits of detection (LOD) and quantification (LOQ) of the heavy metals evaluated by the proposed method using ICP-MS. The level of the studied analytes in a certified reference material (CRM) was determined using the proposed method to check for its accuracy. Since henna and walnut tree bark CRM could not be found, bush branches and leaves CRM from the China National Analysis Center for Iron and Steel were used. Both the blank solutions and CRM were similarly analyzed as the samples.

### 2.5. ICP-MS Determination

The analytical measurements of Pb, Cd and As in the digested henna and walnut tree bark samples were carried out with a Perkin Elmer SCIEX Elan 9000 ICP-MS (Perkin-Elmer SCIEX, Norwalk, CT, USA) after its calibration using the certified stock solutions and optimization according to the recommendation of the manufacturer. The operating conditions of ICP-MS for Pb, Cd, and As determination are provided in Table 2.

### 2.6. Statistical Analysis

The results for heavy metals estimation were tabulated, expressed as mean ± standard deviation (SD), and analyzed for statistical significance using Statistical Package for Social Sciences (SPSS version 23.0). Independent sample *t*-test was used to determine the level of statistical significance between the mean concentration levels of Pb, Cd and As of henna samples and those of walnut tree bark samples; differences at *p* < 0.05 were considered significant.

## 3. Results and Discussion

### 3.1. Analytical Characteristics

A Perkin Elmer method was used for the calculation of the LOD and LOQ values of the studied heavy metals evaluated by the used ICP-MS method [75]. To determine the LOD and LOQ, a blank solution used for the digestion of henna and walnut tree bark samples was first used to blank the instrument before recording the concentrations of Pb, Cd and As in 10 μg·L^−1^ solution of the samples (Table 3). The LOD and LOQ were calculated using the following equations:

LOD = 3 SD_blank_ ⋅ C_sample_/(I_sample_ − I_blank_), (1)

LOQ = 10 SD_blank_ ⋅ C_sample_/(I_sample_ − I_blank_), (2)

where SD_blank_, C_sample,_ I_sample,_ and I_blank_ are the recorded standard deviation for the blank signal, the analyte concentration in the sample [μgL^−1^], the recorded signal intensities for the sample, and the recorded signal intensities for the blank, respectively.

The LOD and LOQ were additionally calculated in the original samples (µg·g^−1^) by considering the amount of digested sample (0.2 g) and the final dilution used during the procedure (100 mL). The results obtained for the LOD and LOQ values of the selected heavy metals in henna and walnut tree bark samples are listed in Table 3. The LOD values of the studied heavy metals were 1, 2 and 5 ng·kg^−1^ for Pb, Cd and As, respectively, and the LOQs were 4, 5 and 30 ng·kg^−1^. The LOD and LOQ values were adequate for the determination of Pb, Cd, and As in henna and walnut tree bark samples.

**Figure 1:**
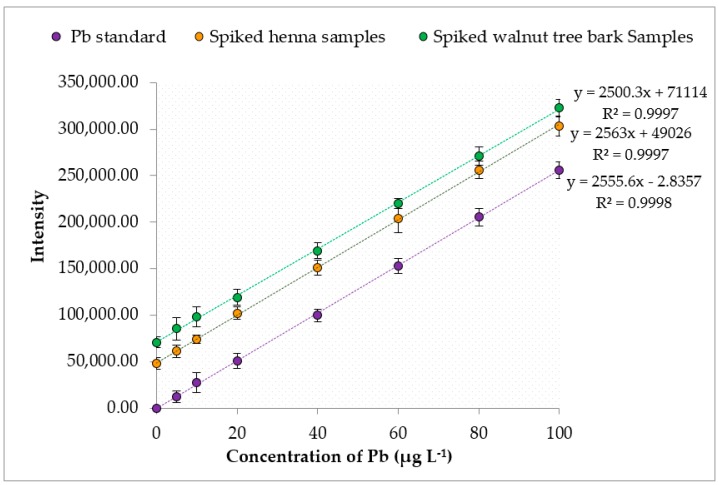
Recoveries of Pb from spiked henna and walnut tree bark samples.

**Figure 2:**
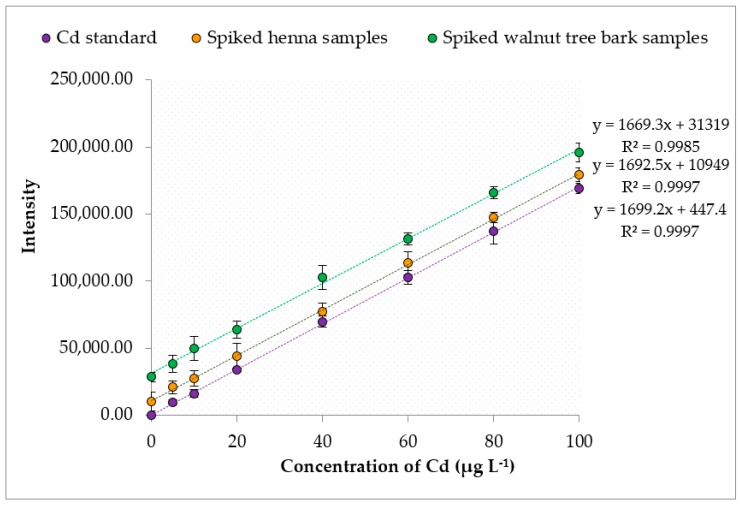
Recoveries of Cd from spiked henna and walnut tree bark samples.

**Figure 3:**
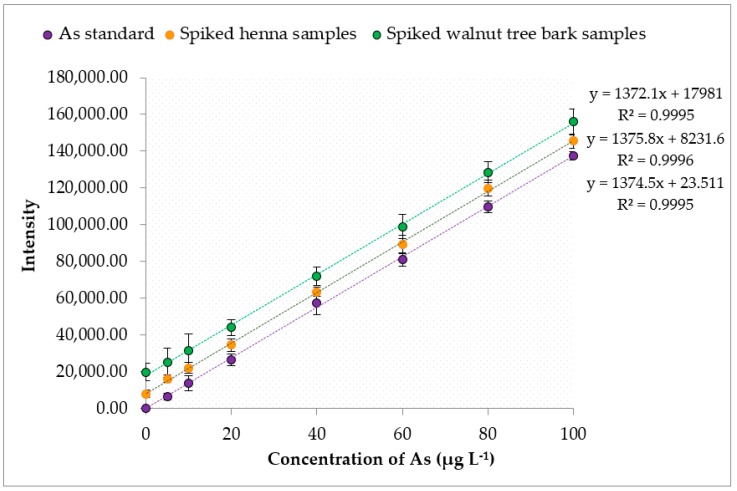
Recoveries of As from spiked henna and walnut tree bark samples.

The results were validated through recovery tests. The recoveries of the studied heavy metals were evaluated by adding Pb, Cd and As standard solutions ranging from 5 µg·L^−1^ to 100 µg·L^−1^ to the henna and walnut tree bark samples. The samples were digested and then analyzed by ICP-MS at optimum conditions. The absolute recovery value for the entire calibration range of each heavy metal was obtained from the ratio between the slope of the line corresponding to the spiked henna or walnut tree bark samples and the slope of the line for direct standard analysis by ICP-MS, as shown in Figure 1, Figure 2 and Figure 3. The obtained recoveries of Pb, Cd and As in the henna samples were 100.3%, 99.6%, and 100.1%, respectively; and in walnut tree bark samples, 97.8%, 99.8% and 98.2 (Table 4). The high recovery values clearly indicated the absence of analyte loss during the sample preparation step and that sensitivity (intensities) was not influenced by the henna and walnut tree bark samples matrix, which allowed us to use a linear calibration technique without needing a standard addition technique.

To evaluate the accuracy of the ICP-MS proposed method, Pb, Cd and As contents in a CRM (NCS DC the CRM 73348-bush branches and leaves) were determined. The CRM was digested and analyzed by the ICP-MS proposed method. The results are summarized in Table 5. The determined values agreed well with those of the certified material, with relative accuracy errors of 1.41, 7.14 and 2.11% for Pb, Cd and As, respectively. This good agreement supported the suitability and accuracy of the proposed ICP-MS method.

### 3.2. Heavy Metals Content in Henna and Walnut Tree Bark Samples

The concentration levels of Pb, Cd and As (µg·g^−1^) in the henna and walnut tree bark samples collected from local herbalist shops in Benghazi markets, Libya are presented in Table 6 and Table 7, respectively. Lead and cadmium were found in all the samples; meanwhile, arsenic was found in all the samples except one henna sample (H6), which had an arsenic content of less than the LOD of 0.0077 µg·g^−1^ (Table 4). Based on the overall mean concentrations, shown in Table 6 and Table 7, the mean heavy metal contents in the henna and walnut tree bark samples were in the following decreasing order: Pb > Cd > As. The lead contents in the henna samples varied from 0.47 ± 0.01 µg·g^−1^ (H10) to 31.98 ± 0.93 µg·g^−1^ (H15), with an overall mean concentration of 15.13 ± 11.51 µg·g^−1^ (Table 6). In the walnut tree bark samples, the values varied from 3.43 ± 0.10 µg·g^−1^ (S9) to 41.47 ± 1.82 µg·g^−1^ (S3), with an overall mean concentration of 23.16 ± 8.89 µg·g^−1^ (Table 7). The cadmium content in the henna varied from 0.29 ± 0.01µg·g^−1^ (H6) to 11.55 ± 0.40 µg·g^−1^ (H10), with an overall mean concentration of 6.30 ± 3.59 µg·g^−1^ (Table 6). In the walnut tree bark, the values varied from 1.41 ± 0.01 (S9) µg·g^−1^ to 15.69 ± 0.24 µg·g^−1^ (S6), with an overall mean concentration of 10.18 ± 4.35 µg·g^−1^ (Table 7). The arsenic contents of the henna samples varied from the LOD of 0.0077 µg·g^−1^ (H6) to 1.45 ±0.04 µg·g^−1^ (H7) with an overall mean concentration of 0.68 ± 0.33 µg·g^−1^ (Table 6). In the walnut tree bark, the arsenic contents varied from 0.26 ± 0.01 µg·g^−1^ (S9) to 2.41 ± 0.07 µg·g^−1^ (S7), with an overall mean concentration of 1.17 ± 0.75 µg·g^−1^ (Table 7).

In accordance to Table 6 and Table 7, it was noted that standard deviations of the overall means concentrations of the studied heavy metals in henna (11.51, 3.59 and 0.33 for Pb, Cd and As, respectively) and walnut tree bark products (8.89, 4.35 and0.75 for Pb, Cd and As, respectively), were very high. The very high standard deviations are not resulted as an error, but rather a significant difference between the concentrations of Pb, Cd and As levels in the different of henna and walnut tree bark products themselves. Indeed, the variation in the concentration levels of heavy metals in the different brands of henna or walnut tree bark products might be attributed to the difference in the way of manufacturing the products (different chemical coloring additives) and product origin, which varies in climatic conditions. It was also observed, the Pb concentration was higher in black henna than in the green henna (See Table 6). However, these results were observed by other investigators [1,11,69] who reported that Pb concentration in black henna is higher than that in green henna.

**Figure 4:**
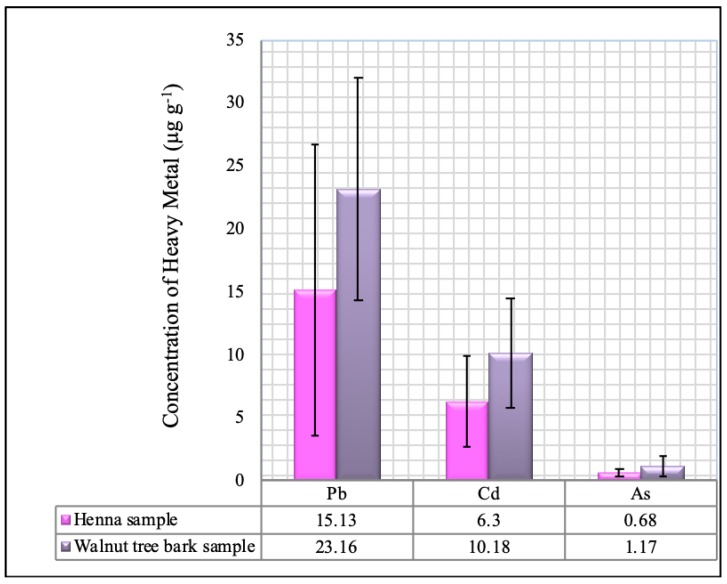
Comparison of mean concentration levels of Pb, Cd and As in henna and walnut tree bark samples.

The results obtained in this study also revealed that the walnut tree bark samples had high levels of heavy metals whereas the henna samples had low levels (Figure 4). Independent sample *t*-test was conducted in order to determine whether this difference between henna and walnut tree bark samples was statistically significant (Table 8). The results indicated that there are significant variations between the concentration levels of heavy metals in henna and in walnut tree bark samples, *p* < 0.05 (Table 8). Given that barks have longer lifespans than leaves, the former had larger heavy metal accumulation than the latter. Moreover, walnut tree bark products are usually stored in uncovered polyethylene containers in contrast to henna products, which are sold in tightly closed polyethylene packages. Thus, walnut bark products may be more exposed to heavy metals than henna products.

### 3.3. Toxicity of Heavy Metals

As shown in Table 6 and Table 7, 60% of the 15 henna samples and 90% of the 10 walnut tree bark samples had Pb levels that are much higher than the limits recommended by the US FDA (20 μg·g^−1^) and Canadian Government (10 μg·g^−1^). Approximately 80% of the 15 henna samples and 90% of the 10 walnut tree bark samples contained Cd levels were much higher than 3 μg·g^−1^ as recommended by the Canadian Government. However, As concentrations in the tested samples were lower than the regulated limit of 3 μg·g^−1^ recommended by the US FDA and the Canadian government (Table 6 and Table 7). The Pb and Cd concentrations in the studied henna and walnut tree bark traditional plant cosmetics were high and pose a major public health hazard especially for the women users. Although the As concentrations in the samples were minimal, the slow release of As may nevertheless have harmful effects on the human body. Thus, the prolonged use of traditional plant cosmetics can increase the absorption of Pb, Cd and As into the human body and elevate the health hazards, especially among women that regularly use henna and walnut tree bark cosmetics.

## 4. Conclusions

The study indicated that Pb and Cd contents in most henna and walnut tree bark products were far above the recommended limits, whereas As concentrations were lower in the most products. It is also noted that there is a very high difference between the concentration levels of Pb, Cd and As in henna and walnut tree bark products themselves. The high difference between the Pb, Cd and As concentrations levels is due to the difference between the samples, sources, and contamination percentage. It is also noted that the walnut tree bark products had higher concentrations of Pb, Cd and As than the henna products, and the differences were statistically significant. Therefore, the walnut tree bark products are more exposed to these heavy metals. They may cause harmful effects to the consumers over time. As Libya currently has no proper safety regulations, strict legislation for enforcing the acceptable limits of potential contaminants in traditional plant cosmetics and good manufacturing practice must be established, and regular inspection of marketed natural products should be conducted for the detection of fraudulent or hazardous products. The selling of unlabeled or noncompliant products should be prohibited, especially henna products because they pose considerable risk to young children and pregnant women. Further studies on traditional plant cosmetics and medicinal plant samples in local markets are highly recommended.

## Figures and Tables

**Table 1:** Microwave digestion program for the digestion of henna and walnut tree bark samples.

Parameters	Steps				
	1	2	3	4	5
**Power (%)**	90	90	0	0	0
**Time (min)**	5	15	10	10	10
**Temp (°C)**	180	180	50	50	50
**Pressure (bar)**	60	60	50	50	50
**Ramp (min)**	36	10	10	10	10

**Table 2:** Operating conditions for ICP-MS determination of Pb, Cd and As in digested henna. and walnut tree bark samples.

Parameters	Conditions
RF Generator	40 MHz
RF Power	1000 W
Spray Chamber	Ryton Scott
Nebulizer	Cross-Flow
Plasma gas flow rate	15.0 L/min
Auxiliary gas flow rate	1.0 L/min
Nebulizer gas flow rate	0.60 L/min
Sampler and skimmer cone	Nickel
Resolution	0.7 ± 0.1 amu
Dwell time	250 ms
Sweeps/Reading	20
Reading/Replicates	3

**Table 3:** The LOD and LOQ of Pb, Cd and As.

Heavy Metal	I_blank_	SD_blank_	I_sample_	LOD (ng·L^−1^)	LOD (ng·kg^−1^)	LOQ (ng·L^−1^)	LOQ (ng·kg^−1^)
**Pb**	20.78	2.27	30,164.88	2	1	7	4
**Cd**	28.04	2.05	16,199.97	4	2	10	5
**As**	43.07	7.14	13,869.12	10	5	50	30

**Table 4:** Recoveries of Pb, Cd and As heavy metals added to henna and walnut tree bark samples.

Heavy Metal	Slope			% Recover	
	Standard Solution	Henna	Walnut Tree Bark	Henna	Walnut Tree Bark
**Pb**	2555.6	2563.0	2500.3	100.3	97.8
**Cd**	1699.2	1692.5	1669.3	99.6	98.2
**As**	1374.5	1375.8	1372.1	100.1	99.8

**Table 5:** ICP-MS determination of Pb, Cd and As in an NCS DC 73,348 bush branches and leaves CRM.

Heavy Metal	Concentration of Heavy (Metal Mean ± SD, μg·g^−1^)		% Relative Error
	Certified Value	Determined Value *	
Pb	7.10 ± 1.10	7.0 ± 1.29	1.41
Cd	0.14 ± 0.06	0.13 ± 0.09	7.14
As	0.95 ± 0.12	0.93 ± 0.20	2.11

* Mean of seven determinations at 95% confidence level. SD = Standard deviation.

**Table 6:** Concentration levels of heavy metals (µg·g^−1^) in the investigated henna samples.

Henna Samples	Color	Place of Origin	Concentration of Heavy Metal (Mean ± SD)		
			Pb	Cd	As
H1	Black	India	20.47 ± 0.39	8.81 ± 0.01	0.56 ± 0.02
H2	Green	Libya	0.81 ± 0.02	6.57 ± 0.15	0.90 ± 0.01
H3	Green	Libya	5.47 ± 0.21	11.48 ± 0.45	0.70 ± 0.01
H4	Green	Libya	1.05 ± 0.02	6.53 ± 0.21	0.40 ± 0.01
H5	Green	Sudan	1.51 ± 0.05	0.40 ± 0.01	0.90 ± 0.04
H6	Green	Sudan	0.85 ± 0.02	0.29 ± 0.01	ND
H7	Black	Sudan	20.51 ± 1.00	4.60 ± 0.01	1.45 ±0.04
H8	Black	Sudan	21.02 ± 0.89	5.32 ± 0.18	0.47 ±0.02
H9	Black	Sudan	22.60 ± 0.83	1.44 ± 0.05	0.72 ± 0.02
H10	Green	Yemen	0.47 ± 0.01	11.55 ± 0.40	0.60 ± 0.02
H11	Black	Yemen	22.51 ± 0.98	6.47± 0.21	1.11 ± 0.04
H12	Black	Pakistan	24.76 ± 0.65	4.88 ± 0.06	0.55 ± 0.01
H13	Black	Pakistan	25.68 ± 0.59	7.34 ± 0.29	0.41 ± 0.01
H14	Black	India	27.23 ± 0.43	7.48 ± 0.32	0.71 ± 0.05
H15	Black	India	31.98 ± 0.93	11.32 ± 0.30	0.67 ± 0.01
Over all mean ± SD (*n* = 15)			15.13 ± 11.51	6.30 ± 3.59	0.68 ± 0.33

ND = Not detected (lower than the LOD for As of 0.008 µg·g^−1^), SD = Standard deviation. SD = Standard deviation.

**Table 7:** Concentration levels of heavy metals (µg·g^−1^) in the investigated walnut tree bark samples.

Walnut Tree Bark Samples	Color	Place of Origin	Concentration of Heavy Metal (Mean ± SD)		
			Pb	Cd	As
S1	Dark brown	Algeria	22.04 ± 1.01	11.65 ± 0.29	0.96 ± 0.03
S2	Dark brown	Algeria	25.73 ± 0.03	13.73 ± 0.03	0.55 ± 0.01
S3	Dark brown	Algeria	41.47 ± 1.82	12.30 ± 0.21	2.25 ± 0.04
S4	Dark brown	Algeria	21.01 ± 0.01	5.33 ± 0.10	1.59 ± 0.05
S5	Light brown	Morocco	20.47 ± 0.55	9.34 ± 0.21	1.78 ± 0.05
S6	Light brown	Morocco	25.18 ± 0.05	15.69 ± 0.24	0.85 ± 0.04
S7	yellowish brown	Tunisia	24.12 ± 1.06	14.45 ± 0.41	2.41 ± 0.07
S8	yellowish brown	Tunisia	21.86 ± 0.58	11.19 ± 0.17	0.44 ± 0.01
S9	Brown	Sudan	3.43 ± 0.10	1.41 ± 0.01	0.26 ± 0.01
S10	Brown	Sudan	26.24 ± 0.50	6.69 ± 0.29	0.61 ± 0.01
Overall mean ± SD (*n* = 10)			23.16 ± 8.89	10.18 ± 4.35	1.17 ± 0.75

SD = Standard deviation.

**Table 8:** Comparative between concentration levels of Pb, Cd and As in henna and walnut tree bark samples.

Element	Concentration of Heavy Metal (µg·g^−1^)		F Value	*p* Value
	Henna Sample	Walnut Tree Bark Sample		
	Mean ± SD	Mean ± SD		
Pb	15.13 ± 11.51	23.16 ± 8.89	18.37	0.001
Cd	6.30 ± 3.59	10.18 ± 4.35	2.233	0.000
As	0.68 ± 0.33	1.17 ± 0.75	47.80	0.002

SD = Standard deviation.
